# The Route to Supercurrent Transparent Ferromagnetic Barriers in Superconducting Matrix

**DOI:** 10.1021/acsnano.9b00888

**Published:** 2019-04-12

**Authors:** Yurii P. Ivanov, Soltan Soltan, Joachim Albrecht, Eberhard Goering, Gisela Schütz, Zaoli Zhang, Andrey Chuvilin

**Affiliations:** † Department of Materials Science and Metallurgy, University of Cambridge, Cambridge CB3 0FS, United Kingdom; ‡ Erich Schmid Institute of Materials Science, Austrian Academy of Sciences, Jahnstraße 12, A-8700 Leoben, Austria; § School of Natural Sciences, Far Eastern Federal University, 690950 Vladivostok, Russia; ⊥ Department of Physics, Faculty of Science, Helwan University, 11792 Cairo, Egypt; ∥ Max-Planck-Institute for Intelligent Systems, Heisenbergstr. 3, D-70569 Stuttgart, Germany; # Research Institute for Innovative Surfaces FINO, Beethovenstr. 1, D-73430 Aalen, Germany; ∇ CIC nanoGUNE Consolider, Av. de Tolosa 76, 20018 San Sebastian, Spain; ○ Basque Foundation for Science, IKERBASQUE, Maria Diaz de Haro 3, 48013 Bilbao, Spain

**Keywords:** high-temperature superconductor, epitaxial oxides, ferromagnetic oxide, SFS junction, quantum electronics

## Abstract

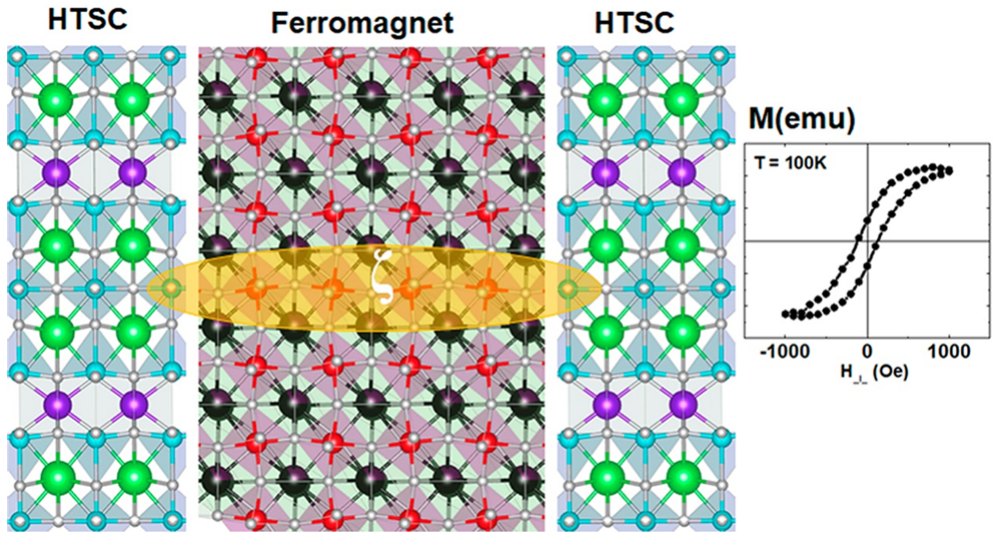

A ferromagnetic barrier thinner than the coherence length in high-temperature superconductors is realized in the multilayers of YBa_2_Cu_3_O_7-δ_ and La_0.67_Ca_0.33_MnO_3_. We used epitaxial growth of YBCO on ⟨110⟩ SrTiO_3_ substrates by pulsed laser deposition to prepare thin superconducting films with copper oxide planes oriented at an angle to the substrate surface. Subsequent deposition of LCMO and finally a second YBCO layer produces a superconductor/ferromagnet/superconductor trilayer containing an ultrathin ferromagnetic barrier with sophisticated geometry at which the long axis of coherence length ovoid of YBCO is pointing across the LCMO ferromagnetic layer. A detailed characterization of this structure is achieved using high-resolution electron microscopy.

The interfaces between the half-metal ferromagnet manganite La_2/3_Ca_1/3_MnO_3_ (LCMO) and the high-temperature superconductor YBa_2_Cu_3_O_7-δ_ (YBCO) are a very motivational subject because of the fundamental questions aiming to reveal relations between crystallographic and electronic structures.^1−19^ The understanding of the interface properties can also aid in potential applications of hybrid oxide superconductor/ferromagnet/superconductor (SFS) structures in superconducting electronics and quantum computing.^20^ So far, it is established that the interaction between thin ferromagnetic and superconducting layers leads to a suppression of either superconductivity or magnetism or both.^21−28^ This is related to the fact that in order to maintain superconductivity, a ferromagnetic layer should be made thinner than a coherence length ζ of the superconductor,^29^ which for optimally doped YBCO is ζ_*c*_ = 0.3 nm in the *c*-direction,^30−34^ a typical growth direction for SFS layers. Deposition of that thin LCMO layer is technically challenging, but what is more important is that it will hardly preserve its ferromagnetic properties at this scale. Though the conditions for superconductivity and ferromagnetism seem to be mutually exclusive, the solution may be found in reorienting the growth direction of YBCO, so that a much longer coherence length in the *ab*-plane ζ_*ab*_ = 1.6 nm could be utilized (see Figure 1).^29,35,36^ Then the thickness of the ferromagnetic barrier may be made up of 2–3 unit cells, which gives hope to preserving ferromagnetism in the layer.^1^ This potentially may be achieved experimentally by the epitaxial growth of YBCO/LCMO/YBCO multilayers on a SrTiO_3_ (STO) substrate with orientation different than (100). It should also be noted that the charge transport across the barrier as well as the magnetic properties of very thin layers can also be affected by the termination atomic plane sequence at the top and bottom interfaces of the ferromagnetic layer due to the rearrangement of the electron orbitals in the interfacing copper oxide planes.^37,38^

**Figure 1:**
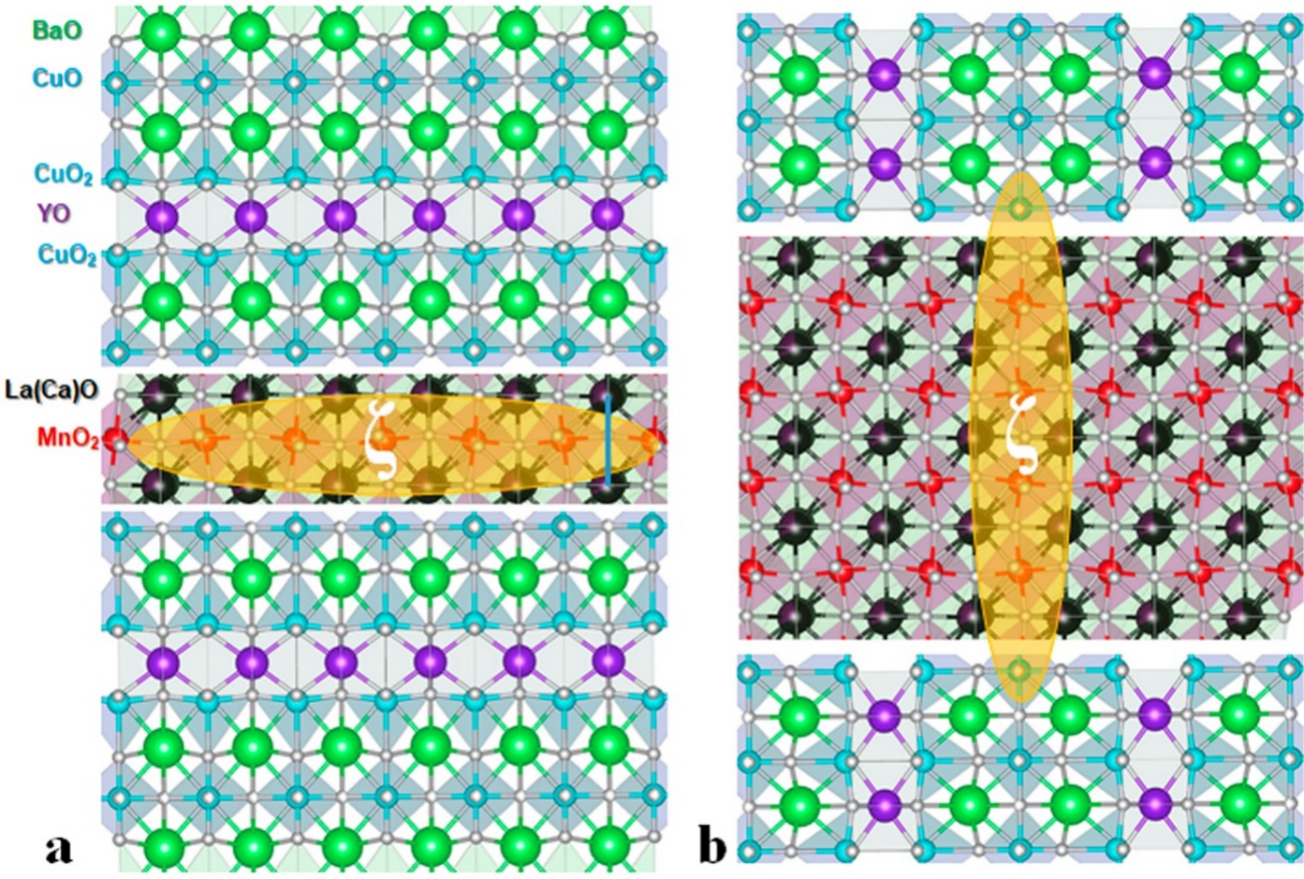
Illustration of the potential increase in the LCMO layer thickness by reorienting of YBCO crystal structure relative to the interface. Yellow ovals represent a coherence length ζ of the YBCO which depends on the orientation of CuO_2_ planes.

The main challenge in this approach is to keep a good interface quality of epitaxial SFS grown on STO substrates oriented differently than (100). The quality of the epitaxial growth and thus the quality of layer interfaces depends on the mismatch between the lattice parameter of the substrate and the film. In the case of the (100)-oriented STO, the mismatch to (001) YBCO is negligible, and typically very high-quality films are obtained with sharp interfaces between the YBCO layers and the ferromagnetic LCMO barrier.^1,38,39^ So far, the conditions for epitaxial growth of YBCO films with *c*-axis oriented in the plane (as schematically shown on the right panel of Figure 1) were not found. In turn, usage of the (110) orientated STO substrate results in polycrystalline (103)/(110)YBCO films with dominant (103) orientation.^40−42^ These films demonstrate a very rough surface which was considered to be unsuitable for the deposition of SFS with a very thin ferromagnetic barrier.

An attempt to deposit an YBCO/LCMO/YBCO trilayer film on STO(110) was reported, without actually showing that the deposition was successful, and the transport measurements were presented, based on which the possibility to realize a supercurrent-transparent ferromagnetic layer was proposed.^29^

In this work, we explore the possibility to grow functional SFS structures on a (110) STO substrate and compare them to common ones grown on (100) STO. We use high-resolution (scanning) transmission electron microscopy (S)TEM and electron energy loss spectroscopy (EELS) to characterize the influence of the STO substrate orientation on the structure of SFS at an atomic level. We provide the direct evidence of the almost perfect trilayer structure of the proposed SFS with a distinct ferromagnetic barrier layer sufficiently thin to transmit the supercurrent.

## Results and Discussion

**Figure 2:**
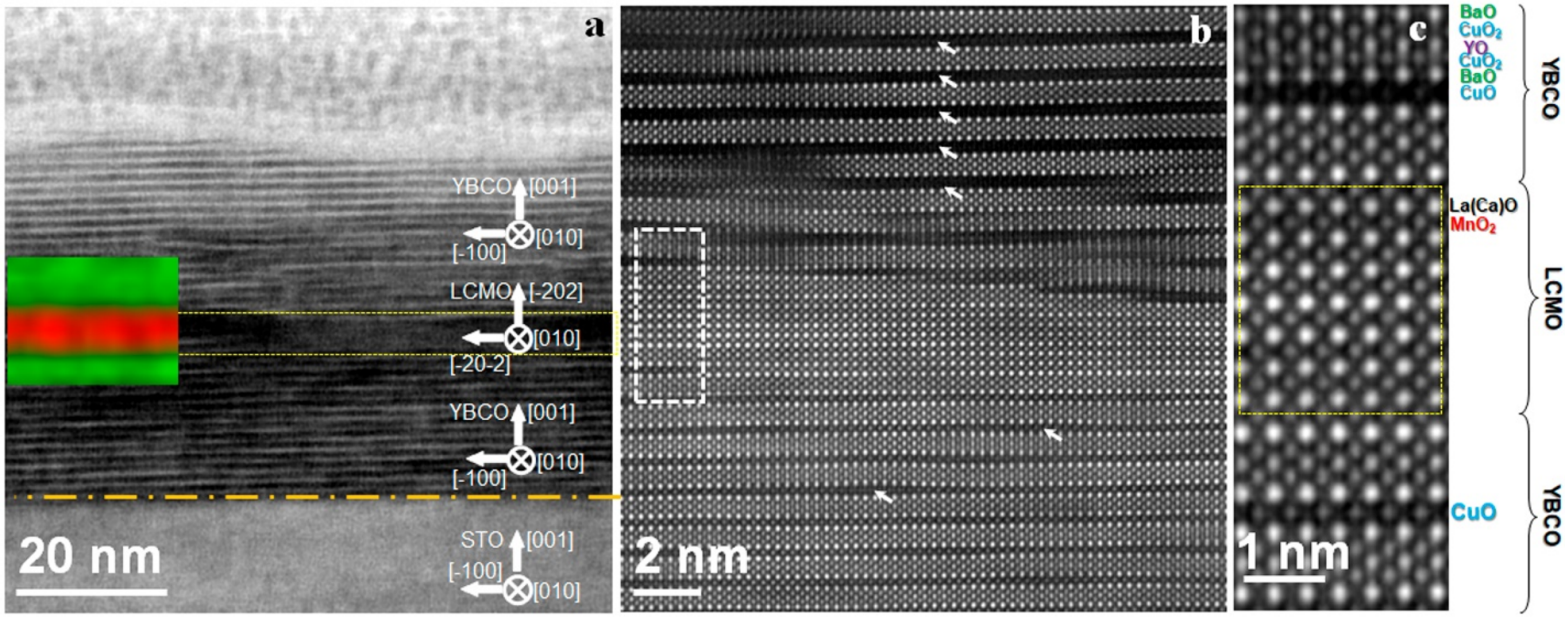
(a) BF STEM overview of the (100)-oriented SFS imaged in a [010] zone axis of YBCO (yellow dashed box marks the position of the ferromagnetic barrier, and brown dashed lines are the interface with STO). EELS elemental map of the ferromagnetic barrier (red color region is from Mn-L_2,3_, green one is from Ba-M_4,5_) is shown in the inset. (b) High-resolution HAADF image of the YBCO layers divided by a ferromagnetic barrier LCMO. (c) Filtered STEM image and reconstructed stacking sequence at the bottom and top interfaces of (100)-oriented SFS (for details see also the Supporting Information).

First, we have characterized the SFS grown on a (100)-oriented STO substrate as the reference sample. As deduced from an overview of the STEM image and EELS map presented on the Figure 2a,c, the structure consists of smooth 20 nm YBCO layers separated by a 2 nm ferromagnetic barrier layer of LCMO. The HR STEM image (Figure 2b) shows that the interfaces are atomically sharp. As is seen from the high-angle annular dark-field (HAADF) image, the atomic stacking sequence at the lower interface LCMO/YBCO is similar to the upper YBCO/LCMO interface (Figure 2d), where CuO_2_ and CuO denote atomic planes with square-planar and linear copper-oxide networks in YBCO. The proposed detailed structure of the interfaces can be found in the Supporting Information. YBCO (100) layers also show a high concentration of YBa_2_Cu_4_O_7_ intergrowths, which are complex defects that can serve as pinning sites for superconducting vortices (the white arrows on the Figure 2b).^39,43,44^ Such complex defects are visible on the HAADF image as the stripes which are wider than the standard CuO rows due to the presence of an additional row of CuO. These intergrowths dominate in the structure of the top YBCO layer as is clearly seen in Figure 2b. The density of intergrowths may vary significantly with the methods used for the film growth and deposition conditions.^44^ It is known that in the composite films, the concentration of these defects is usually much higher.^43,45,46^ It is in agreement with our observation. The introduction of the LCMO layer in between YBCO layers could be a reason for the larger density of the intergrowths in the top YBCO layer.

Thus, in the case of the (100)-oriented substrate, YBCO grows layer by layer in the (001) direction perpendicular to the STO substrate with a fast growth direction (100) in-plane and a slow growth direction (001) out-of-plane of the substrate.

**Figure 3:**
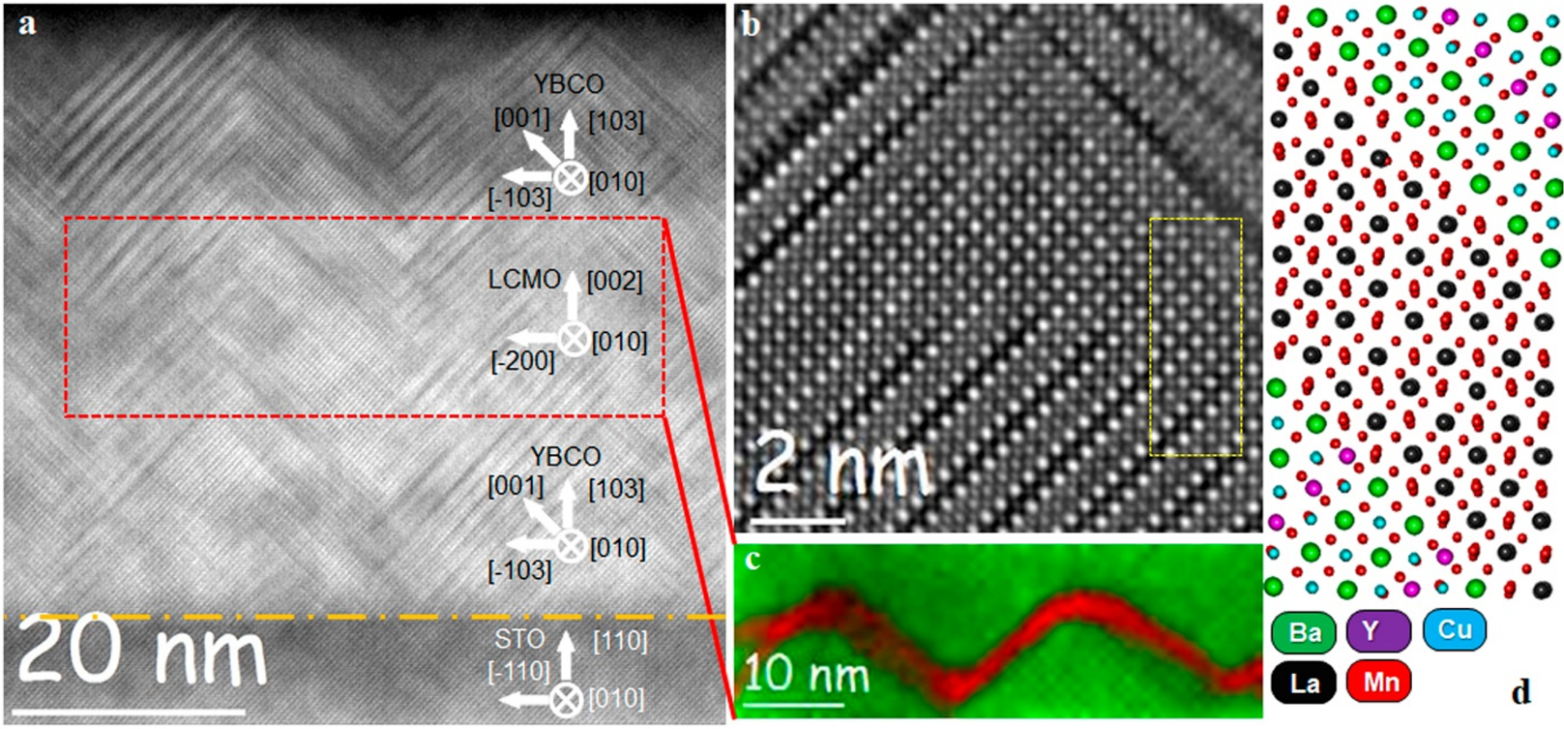
(a) HAADF STEM overview of the (110)-oriented SFS (brown dashed line is the interface with STO). (b) High-resolution HAADF image of the YBCO layers divided by the ferromagnetic barrier LCMO. (c) EELS elemental map of the ferromagnetic barrier (red color region is from Mn-L_2,3_, green one is from Ba-M_4,5_). (d) Stacking sequence at the bottom and top interfaces of (110)-oriented SFS reconstructed from the yellow dashed box in (b); [010] zone axis of YBCO.

The sample with a (110) substrate orientation has a very different picture of epitaxial growth. The most favorable epitaxial mismatch for the growth on the (110) STO substrate is the {103} crystallographic orientation of YBCO, which corresponds to the 45° inclination of the *c*-axis with respect to the substrate surface.^47^ In this case, the fast and slow growth directions are both at 45° to the substrate. The grains nucleate in one of four possible orientations of {103}, leading to a polycrystalline structure with dominating 90° grain boundaries. Due to the high temperature of the substrate, the heterogeneous nucleation rate is very high and the resultant grain size is small. Additionally, due to the inclination of the fast growth direction relative to the substrate surface, these films typically do not grow flat, but rather develop a characteristic pyramidal surface profile. Figure 3 shows the cross section of the SFS grown on the (110)-oriented STO substrate seen in the [010] zone axis of YBCO. Two types of the domains with perpendicular orientation of *c*-axis are clearly seen. The lateral size of the domains is of the order of 10 nm. Since (001) is the slow growth direction of YBCO, four adjacent domains coalesce to form a square pyramid of a height of about 10 nm. These pyramids continue to grow, forming the corrugated surface of the YBCO bottom layer.

Surprisingly, the LCMO barrier layer grown at these deposition conditions on top of the YBCO layer uniformly covers the rough termination surface (see Figure 3), preserving the epitaxial relation with the substrate. As a result of such overgrowth, the LCMO barrier layer in between the YBCO layers forms a triangular wave with the periodicity twice the domain size of YBCO layers, 20 nm. This is proved by the EELS analysis which identifies the LCMO barrier layer with an average thickness of ∼2 nm (see Figure 3c).

**Figure 4:**
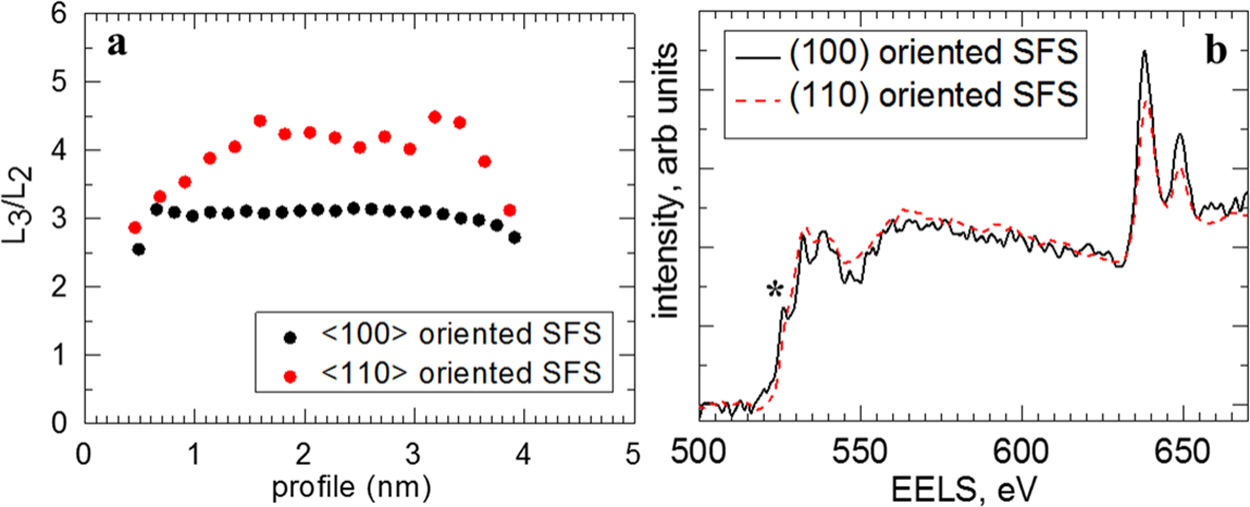
(a) L_3_/L_2_ ratio of the Mn edge measured across the ferromagnetic barrier layer and the (b) average spectra across the layers for the (100)- and (110)-oriented SFS.

The oxidation state of 3d transition metals can be locally determined by analyzing the intensity ratio of the white lines of a (in particular for Mn) L edge in EELS spectra.^48^ Supporting evidence can be obtained from the analysis of the fine structure of the O K edge at the prepeak position, which is sensitive to the valence state of the nearest-neighbor 3d transition-metal ions.^49^ The calculation of Mn L_3_/L_2_ ratio was done by using a two step function as described in ref (48). Figure 4a shows the typical dependence of the Mn L_3_/L_2_ ratio across the ferromagnetic barrier for samples of (100)- and (110)-oriented SFS. Overall, the L_3_/L_2_ ratio for (110) SFS is substantially higher with respect to (100) SFS, indicating a decreased oxidation state of Mn ions in the LCMO layer for (110) SFS. This is in agreement with the observed vanishing of the prepeak at the O K edge (marked by a star on the Figure 4b) for (110)-oriented SFS.

The profile across the LCMO barrier layer of the Mn L_3_/L_2_ ratio for the (100)-oriented SFS is very smooth and shows a very similar value for different areas probed by EELS mapping. In contrast, the profile for the (110)-oriented SFS shows more irregularities and a larger variation of the Mn L_3_/L_2_ ratio across the layers. These differences may be attributed to substantially different interface structures in these two cases, in particular, different ways the CuO_*x*_ planes are connected to the interface, which determines different a charge transfer across the interface.^37,50^ The proposed detailed structure of the interfaces can be found in the Supporting Information.

**Figure 5:**
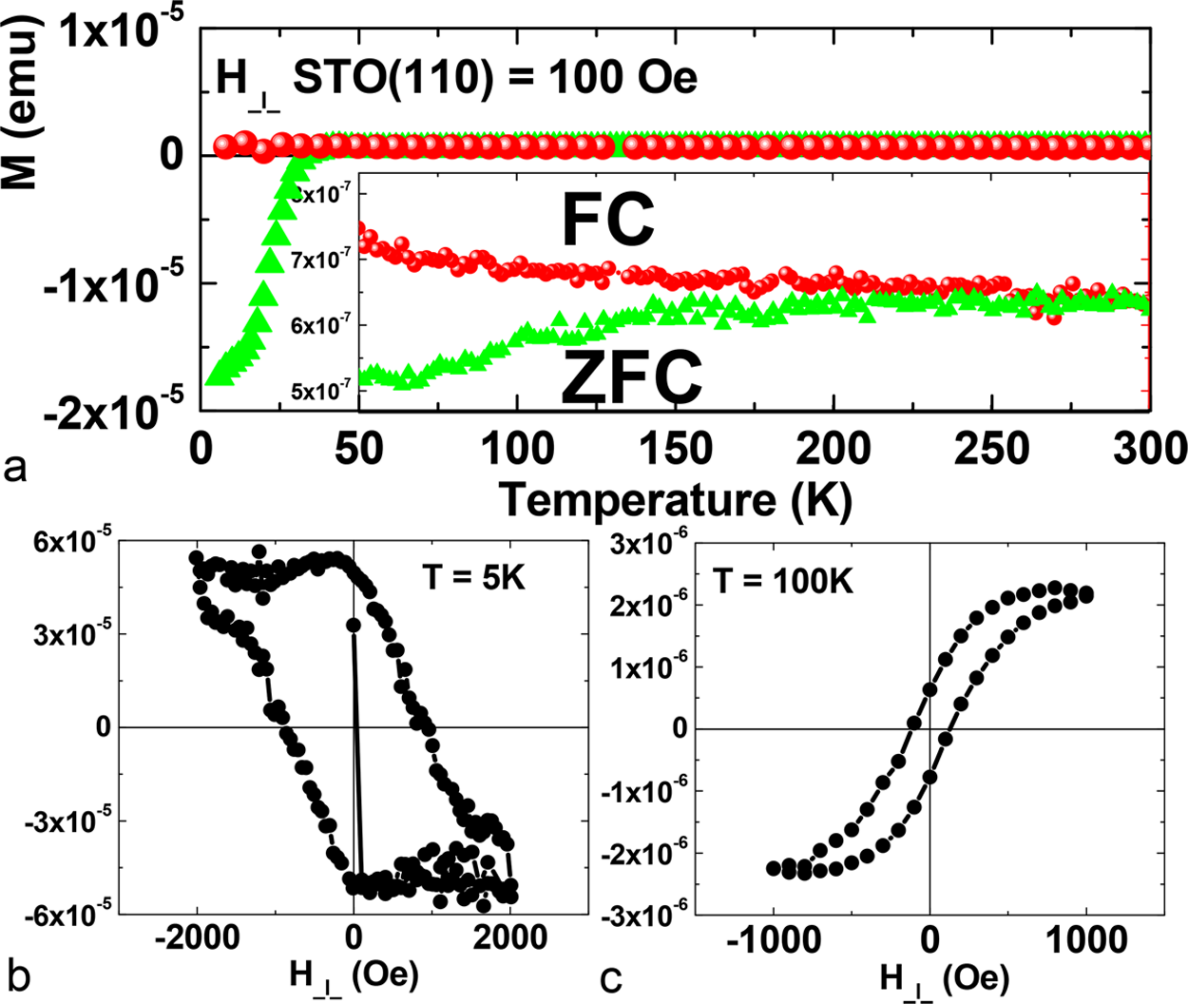
Magnetization *versus* temperature *M*(*T*) (a) and magnetic loops *M*(*H*) at *T* = 5 K (b) and *T* = 100 K (c) for YBCO/LCMO/YBCO on STO(110) with *t*_LCMO_ = 2 nm. The external magnetic field *H* is aligned in-plane of the sample in all cases (a–c).

The remining question is if this thin LCMO layer is still ferromagnetic. Magnetization *versus* temperature curves for the (110) SFS sample were measured in an external field of *H* = 100 Oe parallel to the film plane in zero-field cooled (ZFC) and field-cooled (FC) regimes using a SQUID magnetometer (see Figure 5a). The ZFC curve shows a strong diamagnetic signal below *T* = 30 K, which corresponds to the superconducting transition of YBCO. The inset shows a magnification of the magnetic signal at higher temperatures. It can be seen that the ZFC and FC curves are separated below ∼250 K, indicating the ferromagnetic ordering of the ultrathin LCMO layer. It is known that the Curie temperature is strongly reduced in thin LCMO layers deposited on the STO substrate.^51^ Nevertheless, the 4–5 unit cell thick LCMO layer sandwiched in YBCO shows a *T*_C_ > 200 K. This agrees with previous works^25,33^ which showed that a 7 u.c. thick LCMO layer in between (001) YBCO layers exhibit *T*_C_ = 210 K.

Figure 5b,c shows the magnetization *versus* external magnetic field curves for (110)-oriented YBCO/LCMO/YBCO heterostructures measured at *T* = 5 K and at *T* = 100 K. At 5 K, far below the superconducting transition of the YBCO layers, there is an irreversible behavior, characteristic for high-*T*_C_ superconducting epitaxial YBCO films with strong pinning of Abrikosov vortices. This is in-line with the STEM data which indicate the high density of intergrowth defects, proposed sources for the pinning of superconducting vortices. At 100 K, above the superconducting transition of YBCO and below the Curie temperature of the LCMO layer, the hysteresis loop is typical for a ferromagnetic material.

Assuming the observed structural quality of the layers and interfaces and the desired orientation of CuO_2_ planes to superconductor/ferromagnet interface in (110)-oriented SFS, one could expect the appearance of the supercurrent across a nanoscale ferromagnetic barrier in this system. Indeed, in a previous paper, we reported the experimental evidence of the supercurrent transport across a ferromagnetic barrier for similar (110) SFS structures.^29^ The effect was more pronounced in the case of patterned microscale junctions. This can be explained by the existence of a small number of randomly distributed nanosized pinholes, as shown in the Supporting Information. The estimated surface area ratio of the pinholes is only about 1%, so the effect of the pinholes on the transport measurements could be significantly reduced by micropatterning.

## Conclusions

It has been shown that YBCO/LCMO/YBCO SFS trilayers can be grown on a (110) STO substrate. YBCO layers in this case have a multidomain structure and a rough surface. As a consequence, the LCMO layer has a peculiar complex topography, yet a good uniformity in thickness and perfect interfaces both on the bottom and on the top YBCO layers. Orientation of CuO layers of YBCO normal to a 2 nm thin LCMO layer explains the transparency of (110)-oriented ferromagnetic layer for the supercurrent, which was experimentally shown for this system earlier. The EELS study revealed the difference in the Mn oxidation states across the LCMO barrier layer depending on the crystal growth orientation. Magnetic measurements confirmed the preservation of the ferromagnetic nature of the 2 nm LCMO layer buried in YBCO. The studied (110)-oriented SFS is a good candidate for realizing a high-*T*_C_ SFS junction with a ferromagnetic layer thinner than the coherence length of a high-*T*_C_ superconductor after optimizing the geometry of the devices, in particular their sizes.

## Methods

For both substrate orientations, trilayer YBCO/LCMO/YBCO structures were grown by pulsed laser deposition (PLD). Twenty nm of YBCO was deposited directly on STO substrates, followed by 2 nm of LCMO and of 20 nm of YBCO top layer. All layers were deposited at a 730 °C substrate temperature. Full oxygenation was achieved by post-annealing of the samples at 530 °C and 10^5^ Pa of oxygen pressure for 30 min, followed by slow cooling to room temperature. The cross sections for (S)TEM studies were prepared using a standard focused ion beam (FIB) protocol.^52^ Experiments were carried out on a Titan 60-300 TEM (FEI Company) equipped with a high-brightness field-emission gun (X-FEG), monochromator, and image-side C_S_-corrector (CEOS) and operated at 300 kV. EELS experiments were performed with a post-column EEL spectrometer (Quantum GIF Gatan). The optical conditions of the microscope for the STEM EELS spectrum imaging were set to obtain a probe size of 0.14 nm, with a convergence semi-angle of 10 mrad and collection semi-angle of 12 mrad.

## Supporting Information Available

Additional information and figures on the EDX, EELS, and nanodiffraction study of the (100) and (110) SFSs as well as the results of the high-resolution ABF and HAADF study of the interfaces between the LCMO and YBCO layers (PDF)
