# Supplementary material for: The Route to Supercurrent Transparent Ferromagnetic Barriers in Superconducting Matrix
Source: ACS Nano. 2019 Apr 12;13(5):5655–61. doi: 10.1021/acsnano.9b00888 (PMC8830211; doi:10.1021/acsnano.9b00888)
Supplement: Supplementary file 1 — nn9b00888_si_001.pdf [file nn9b00888_si_001.pdf]

Supporting Information

The Route To Supercurrent Transparent Ferromagnetic Barriers In Superconducting Matrix

Yurii P. Ivanov[†,|,*], *Soltan Soltan*[§,‖], *Joachim Albrecht*[⊥], *Eberhard Goering*[§], *Gisela Schütz*[§], *Zaoli Zhang*[|], *Andrey Chuvilin*[‡,#]

[†] Department of Materials Science & Metallurgy, University of Cambridge, Cambridge CB3 0FS, UK.

[|] Erich Schmid Institute of Materials Science, Austrian Academy of Sciences, A-8700 Leoben, Jahnstraße 12, Austria

[*] School of Natural Sciences, Far Eastern Federal University, 690950, Vladivostok, Russia

[§] Max-Planck-Institute for Intelligent Systems, Heisenbergstr. 3, D-70569 Stuttgart, Germany

[‖] Department of Physics, Faculty of Science, Helwan University, 11792 Cairo, Egypt

[⊥] Lab for intelligent and innovative surfaces, Aalen University - Beethovenstr. 1, D-73430 Aalen, Germany

[‡] CIC nanoGUNE Consolider, Av. de Tolosa 76, 20018, San Sebastian, Spain

IKERBASQUE, Basque Foundation for Science, Maria Diaz de Haro 3, 48013, Bilbao, Spain

KEYWORDS high temperature superconductor, epitaxial oxides, ferromagnetic oxide, SFS junction, quantum electronics

*e-mail: ivanov.yup@gmail.com

EDX study

The image acquisition and spectroscopic analysis was conducted using a FEI Tecnai Osiris with field emission gun TEM/STEM operated at 200 keV, equipped with Super-X windowless EDX detector. The probe size was around 0.2 nm.

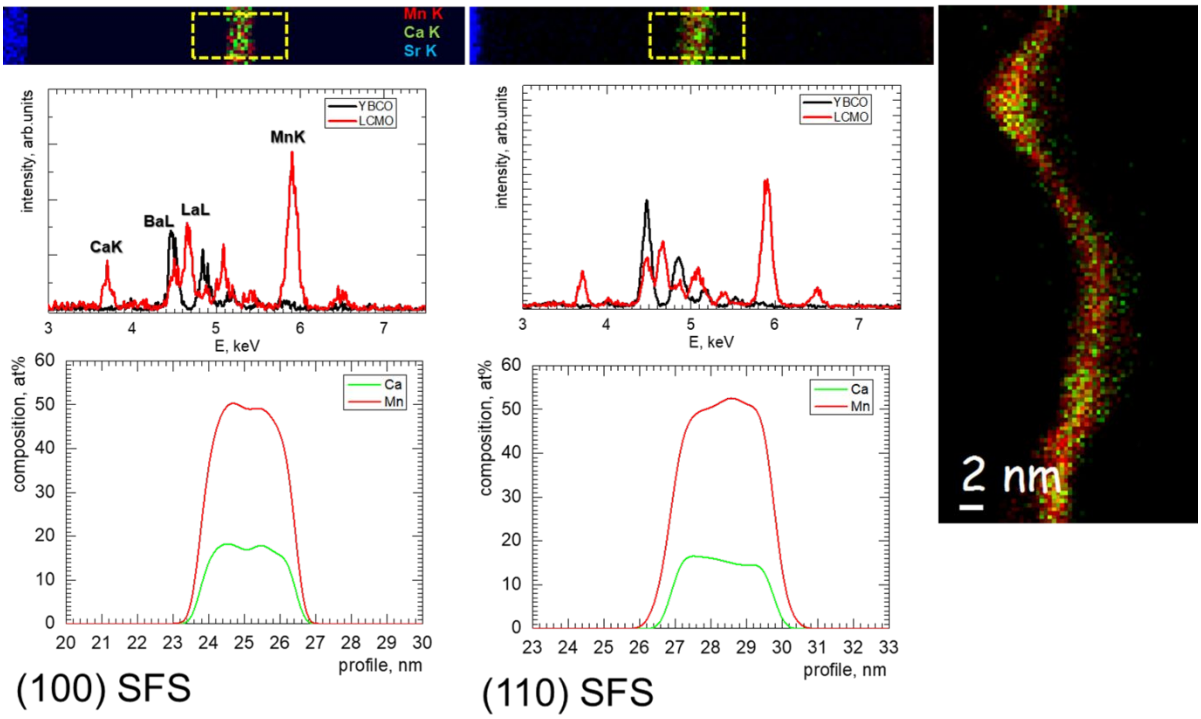

Figure S1. EDX maps across the interface for (100) and (110) SFS. At the bottom the average spectra extracted from YBCO and LCMO layers and the profiles averaged across the yellow box are shown. On the right the larger map of the (110) SFS are presented

The Figure S1 shows the position and composition of the LCMO barrier layer in (100) and (110) SFS oriented SFS. There is no pronounced separation between Ca and Mn. The relative composition extracted from the spectra shown on the Figure S1 is 34±4, 16±4, 51±4 at % for La, Ca and Mn accordingly. It agrees very well with the expected composition of $L_{2/3}Ca_{1/3}MnO_3$ layer.

Nano diffraction study

It is difficult to access separated diffraction patterns of YBCO and LCMO layers by selected area electron diffraction due to its nanoscale size. Thus, we recorded the nano-diffraction patterns using nano probe with the small convergences angle of 1.5 mrad.

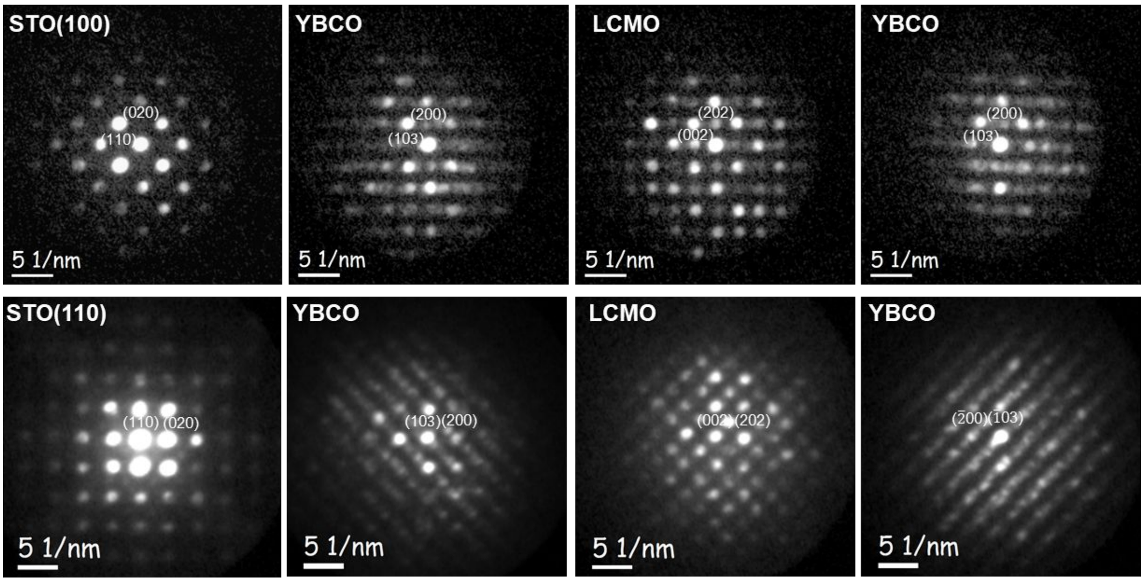

Figure S2. Nano diffraction patterns from STO substrate, YBCO layers and LCMO layer for (100) top and (110) SFS bottom at [100] zone axis of the substrate. The corresponding indexed atomic planes are shown.

As clearly seen from the Figure S2 the layers grow epitaxially. The indexed planes correspond to STO – space group Pm-3m (221) with (200) 0.195 nm and (110) 0.276 nm, YBCO - Pmmm (47) with (200) 0.193 nm and (103) 0.274 nm and LCMO Pnma (62) with (202) 0.193 nm and (200) 0.272 nm.

ABF and HAADF study of the LCMO/YBCO interfaces

To evaluate the possible atomic structure at the LCMO/YBCO interfaces we perform high resolution STEM study using a Titan 80-300 TEM/STEM with field emission gun operated at 300 keV, equipped with probe corrector. The probe size was less than 0.1 nm. For HAADF imaging the collection angle was 54 – 200 mrad. For the ABF imaging the detector angles were adjusted to 10-20 mrad.

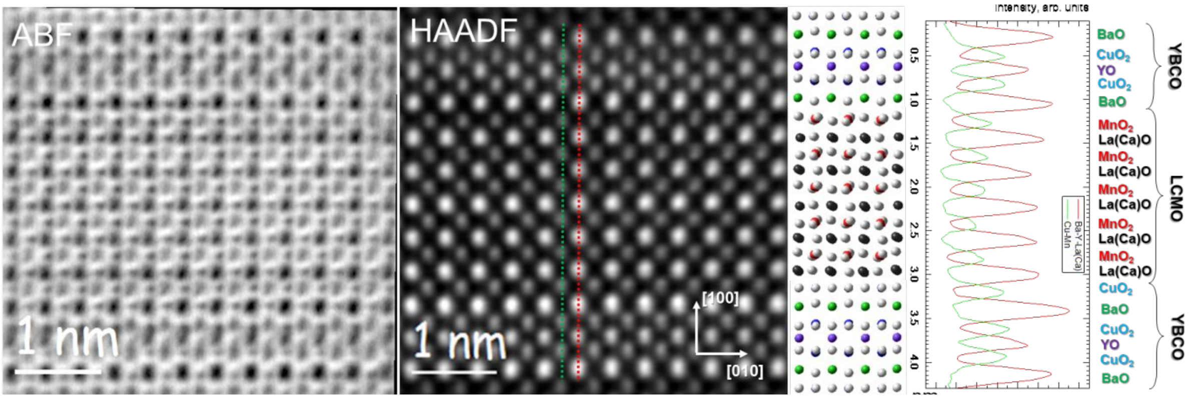

Figure S3. ABF and HAADF image of the YBCO/LCMO/YBCO interfaces for (100) oriented SFS. The possible atomic planes sequence at the interfaces was reconstructed based on the intensity of the atomic columns shown on the right. The intensity profiles correspond to the green and dashed lines on the HAADF image.

Figure 3S shows the HR STEM images of the YBCO/LCMO/YBCO interfaces for (100) oriented SFS. The intensity of the atomic columns in the HAADF image is proportional to the Z number of the corresponding atomic species. The profiles extracted from green and red lines on the HAADF image allows us to propose the possible atomic sequence at the interfaces (Figure S3). For bottom interface it is BaO-CuO2-Y-CuO2-BaO-CuO2-La(Ca)O-MnO2-La(Ca)O. For the top interface La(Ca)O-MnO2- BaO-CuO2-Y-CuO2-BaO. The advantage of the ABF image is the visualization of the atomic columns of the light elements like oxygen. The intensity of the oxygen columns is not varying significantly across the interface. Note it is very difficult to monitor the small oxygen variation by ABF images. A slight local misorientations of the lattice have a giant effect on the ABF contrast in contrary to the HAADF contrast. It is in agreement with recent systematic study of the ABF method.[1]

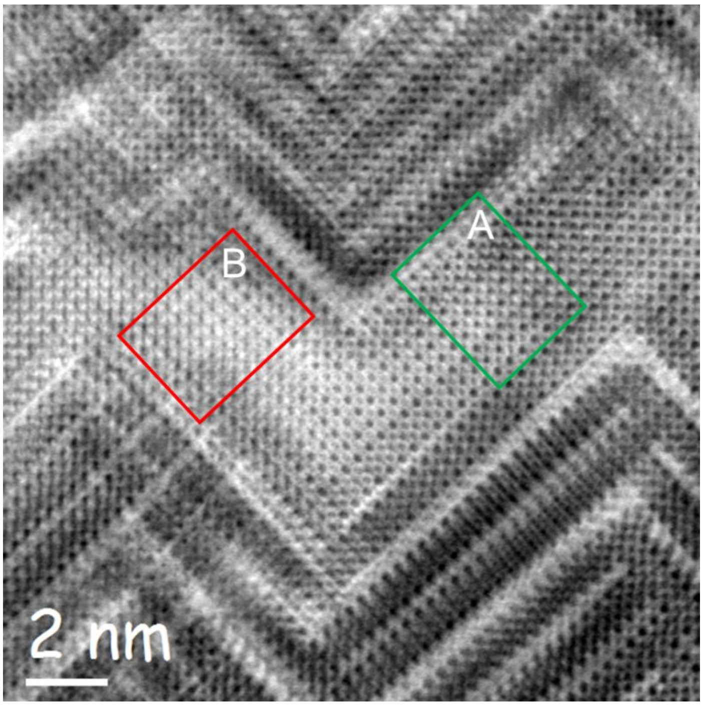

Figure S4. ABF image of the YBCO/LCMO/YBCO interfaces for (110) oriented SFS. The two possible types of the interface structure are marked by green (A) and red rectangles.

Figure S4 shows the ABF image of the (110) SFS. There are at least two possible type of the LCMO/YBCO interface structure. The one A is similar to the case of (100) oriented SFS and another one B is discussed on the Figure S5.

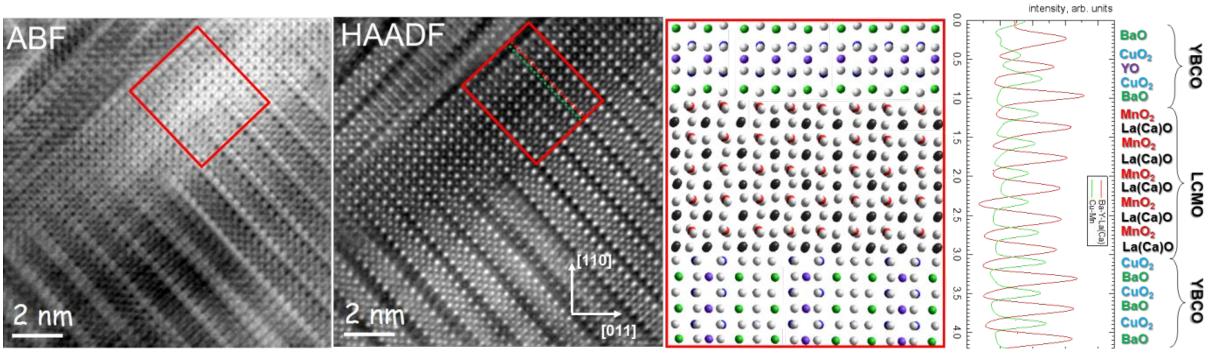

Figure S5. ABF and HAADF image of the YBCO/LCMO/YBCO interfaces type B for (110) oriented SFS. The possible atomic planes sequence at the interfaces was reconstructed based

on the intensity of the atomic columns shown on the right. The intensity profiles correspond to the green and dashed lines on the HAADF image.

Figure S5 shows the HR STEM images of the YBCO/LCMO/YBCO interfaces type B for (110) oriented SFS. The profiles extracted from green and red lines on the HAADF image allows us to propose the possible atomic sequence at the interfaces (Figure S5).

Pinholes in ferromagnetic barrier layer of (110) oriented SFS

We have analysed the continuity of the LCMO barrier layer. In the case of the (100) oriented SFS the LCMO barrier layer is continuous of uniform thickness. In contrary, we observed several small pinholes on the barrier for (110) grown SFS as shown on the EELS spectrum images, Figure S6. The lateral size of pinholes is comparable with the LCMO nominal barrier layer thickness.

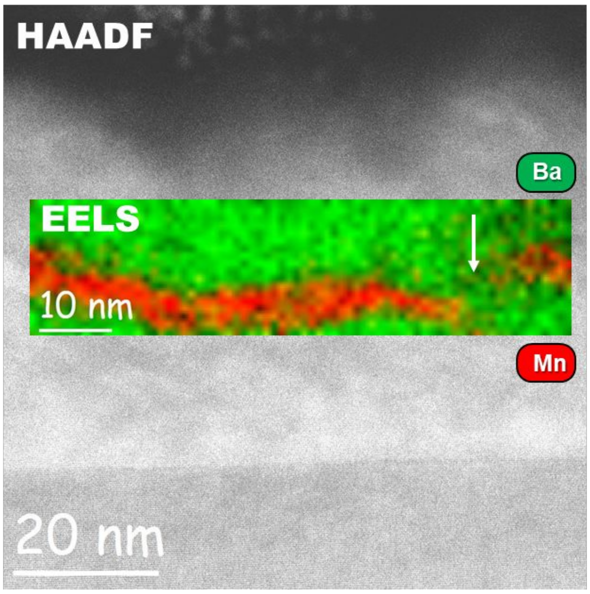

Figure S6. HAADF images and corresponding EELS mapping of the LCMO barrier layer for (110) oriented SFS. The positions of the pinhole in barrier layer are marked by arrow.

References

(1) Gao, P.; Kumamoto, A.; Ishikawa, R.; Lugg, N.; Shibata, N.; Ikuhara, Y. Picometer-Scale Atom Position Analysis In Annular Bright-Field STEM Imaging. *Ultramicroscopy* **2018**, *184*, 177–187.
